# ﻿*Columneagolondrinensis* (Gesneriaceae), a new species from Cerro Golondrinas in the northern Andes of Ecuador

**DOI:** 10.3897/phytokeys.253.144114

**Published:** 2025-03-04

**Authors:** John L. Clark

**Affiliations:** 1 Marie Selby Botanical Gardens, 1534 Mound St., Sarasota, FL 34236 USA Marie Selby Botanical Gardens Sarasota United States of America

**Keywords:** Chocó, Colombia, *Columnea*, Ecuador, Gesneriaceae, taxonomy

## Abstract

Exploratory field expeditions to the Chocó forests in the northern Andes of Ecuador resulted in the discovery of a new species of *Columnea* (Gesneriaceae). *Columneagolondrinensis* J.L.Clark, **sp. nov.**, is described as a narrow endemic from the cloud forests of Cerro Golondrinas in the Carchi Province near the northern Ecuadorian border with Colombia. The oval leaves with a rust-colored multicellular hispid indumentum, terrestrial subwoody habit and deeply bilabiate dark purple corollas with glandular trichomes differentiate this taxon from all other congeners. Based on IUCN guidelines, a preliminary conservation status of Vulnerable (VU) is provided for *C.golondrinensis*.

## Abstract (es)

Las expediciones de campo exploratorias a los bosques del Chocó en los Andes del norte de Ecuador resultaron en el descubrimiento de una nueva especie de *Columnea* (Gesneriaceae). *Columneagolondrinensis* J.L.Clark, **sp. nov.**, se describe como una endémica restringida a los bosques nublados del Cerro Golondrinas, en la provincia de Carchi, cerca de la frontera norte de Ecuador con Colombia. Las hojas ovaladas con un indumento híspido multicelular de color óxido, el hábito subleñoso terrestre y las corolas profundamente bilabiadas de color púrpura oscuro con tricomas glandulares diferencian a este taxón de todos sus congéneres. Según las directrices de la UICN, se proporciona un estatus preliminar de conservación de Vulnerable (VU) para *C.golondrinensis*.

## ﻿Introduction

The Gesneriaceae family, part of the order Lamiales, comprises more than 3,900+ species across 150 genera (Weber 2004; Weber et al. 2013, 2020; GRC 2025). The family is organized into three subfamilies and seven tribes, each representing monophyletic lineages (Weber et al. 2013, 2020; Ogutcen et al. 2021). In the New World, the majority of species belong to the subfamily Gesnerioideae, encompassing over 1,200 species and 77 genera (Clark et al. 2020). Within this group, *Columnea* L. is classified in the tribe Gesnerieae and the subtribe Columneinae (Weber et al. 2013, 2020). A defining generic character of *Columnea* is indehiscent berries, which contrast with the fleshy, bivalved capsules of closely-related genera.

*Columnea* ranges from Mexico south to Bolivia and is most diverse in the northern Andes of Colombia and Ecuador. With over 220 species (Clark et al. 2020; GRC 2025), *Columnea* is the largest genus in the subfamily Gesnerioideae (Weber et al. 2013, 2020). *Columnea* is strongly supported as a monophyletic genus, based on molecular phylogenetic studies (Smith et al. 2013; Schulte et al. 2014). Most subgeneric ranks are artificially defined and not supported by phylogenetic studies (Smith and Carroll 1997; Smith 2000; Clark and Zimmer 2003; Clark et al. 2006; Clark et al. 2012; Smith et al. 2013; Schulte et al. 2014). Thus, the new species is not classified or assigned to a subgeneric rank.

## ﻿Materials and methods

Plants were photographed in the field and subsequently pressed and dried. Specimens are currently deposited at the herbarium at the Pontificia Universidad Católica del Ecuador (QCA). Additional specimens will be distributed to the Field Museum (F), Conservatoire et Jardin Botaniques de la Ville de Genève (G), Missouri Botanical Garden (MO), Marie Selby Botanical Gardens (SEL), New York Botanical Garden (NY), and the United States National Herbarium (US). Photographs were taken of live specimens in the field using a Nikon D100 DSLR with a Nikon 105 mm lens. Morphological observations and measurements were made from live collections, alcohol-preserved material, and digital images using the ImageJ program (https://imagej.nih.gov/ij/).

The extinction risk was assessed following the IUCN Red List Categories and Criteria (IUCN 2012) and updated criteria in the IUCN Standards and Petitions Committee (IUCN 2024). Field observations and collection sites from fieldwork were used to evaluate the IUCN category. The extent of occurrence (EOO) and area of occupancy (AOO) were calculated using the software program GeoCAT (Bachman and Moat 2012) with the default setting of 2 km, which is a 4 km^2^ grid cell.

## ﻿Taxonomic treatment

### ﻿ Columnea golondrinensis

Taxon classificationPlantaeLamialesGesneriaceae

J.L.Clark sp. nov.

FFE12FEB-FEA3-51FD-960B-FCB70ED94C93

urn:lsid:ipni.org:names:77357320-1

Fig. 1

#### Type.

Ecuador. • Carchi: cantón Tulcan, parroquia Chical, Cerro Golondrinas, ridgeline(s) between campsite #1 (sector Río Verde) to campsite #2 (La Laguna), 0°52'20.07"N, 78°12'25.61"W, 1800–2225 m alt., 26 Jan 2024, *John L. Clark, Luis Micanquer, Milton Cantincuz, Mia Johnson & Nolan Exe 18185* (holotype: QCA; isotypes: F, G, MO, NY, SEL, US).

#### Diagnosis.

Vegetatively similar to *Columneasuffruticosa* J.F. Sm. & L.E. Skog due to the presence of ovate leaves with a rust-colored multicellular hispid indumentum, but differing in the terrestrial habit (vs. epiphytic habit in *C.suffruticosa*) and deeply bilabiate corolla (vs. uniformly tubular corolla in *C.suffruticosa*). The deeply bilabiate corolla, dark purple corolla tube, and glandular trichomes throughout the upper and lower lobes are unique characters not found in any other known species of *Columnea*.

#### Description.

Terrestrial subshrub with dorsiventral shoots, 1.0–1.5 m tall, stems green with densely pilose rust to gold-colored multicellular hispid indumentum; internodes 1.0–2.5 cm long. Petioles 1.0–2.5 cm long, red, with densely pilose to gold-colored multicellular hispid indumentum; leaves opposite, pairs strongly anisophyllous, larger leaf 9.0–15.0 cm long, 4.0–7.0 cm wide, ovate-elliptic, apex acuminate, base rounded and slightly oblique, lateral veins 5–9 per side, adaxially light-green, with multicellular hispid indumentum, abaxially uniformly-red, with multicellular hispid indumentum, more densely pubescent on veins, margin entire; smaller leaf 1.0–2.0 cm long, 0.9–1.5 cm wide, lateral veins 2–3 per side, petiole 1–2 mm long, otherwise similar to larger leaf. Inflorescence reduced to 1–3 axillary flowers; bracts triangular, green, 2–3 mm at base, apex broadly acuminate. Pedicels 1.0–1.8 cm long, dark red, densely pilose with multicellular rust-colored hispid indumentum. Calyx lobes uniformly green or green suffused with red, 1.0–1.7 cm long, 0.5–0.7 cm wide at base, oblong, apex acuminate, exterior pilose, with multicellular rust-colored hispid indumentum, interior glabrous, margin mostly entire or with 1–3 serrations. Corolla 5.0–6.2 cm long, 2.0 cm at widest (apex) point, deeply bilabiate, lower lobe recurved, 1.8–2.2 cm long, 3–4 mm wide, lateral and upper lobes fused into a hood, lateral lobes reflexed, rounded, 5 mm at base with acuminate apex, upper lobes fused, 1.0 cm wide, 1.3 cm long, apex bilobed, each lobe rounded, densely pubescent with multicellular rust-colored trichomes, interior uniformly dark purple, covered with glandular trichomes, occasionally with yellow margins, especially along the lower lobes, outer surface dark purple to dark purple suffused with yellow. Filaments ca. 3.5 cm long, connate at base for 0.3 cm and adnate to corolla, anthers ca. 3.0 mm long, 3.0 mm wide, included in the corolla throat, quadrangular. Ovary ca. 4.0 mm long, conical, glabrescent; style 3.5–4.0 cm long, glabrescent, stigma rounded. Nectary comprised of one large dorsal and two smaller lateral glands. Fruit not observed.

#### Phenology.

Collected in flower in January.

#### Etymology.

The specific epithet reflects the type locality, Cerro Golondrinas, where this species is presumably endemic.

#### Distribution and preliminary assessment of conservation status.

*Columneagolondrinensis* is only known from a single population on a ridgeline in Cerro Golondrinas. The region is protected by the recent acquisition and purchase of forest by Fundacion EcoMinga. The forest corresponds to the Chocó Biogeographic Region for the relatively high levels of precipitation and epiphytic diversity. Based on the available information and according to the IUCN Red List criteria (IUCN 2012; IUCN Standards and Petitions Committee 2024), *C.golondrinensis* is preliminarily assessed as Vulnerable (VU) based on a limited area of occupancy (IUCN criterion D2 where AOO < 20 km^2^) and limited number of locations (< 5).

#### Comments.

Most *Columnea* are epiphytic and terrestrial with primary shoots that are characterized as erect, horizontal, dorsiventral (associated with facultative epiphytes), or pendent. The species described here was observed as a multibranched terrestrial subshrub with dorsiventral shoots. It was not observed growing epiphytically and was only observed on a ridgeline with a low canopy and low-growing shrubs where epiphytic diversity was lower. Thus, surrounding forests with higher canopies could host *C.golondrinensis* as an epiphyte.

Several species of *Columnea* have purple-brownish coloration on the corolla tube, but these are consistently mostly yellow with narrow bands of horizontal purple-brownish stripes (Fig. 2). In contrast, the corolla color in *C.golondrinensis* is mostly dark purple to brown with yellow margins on the lower lip. The corolla colors in *C.golondrinensis* are unique amongst all known members of *Columnea*.

**Figure 1.**
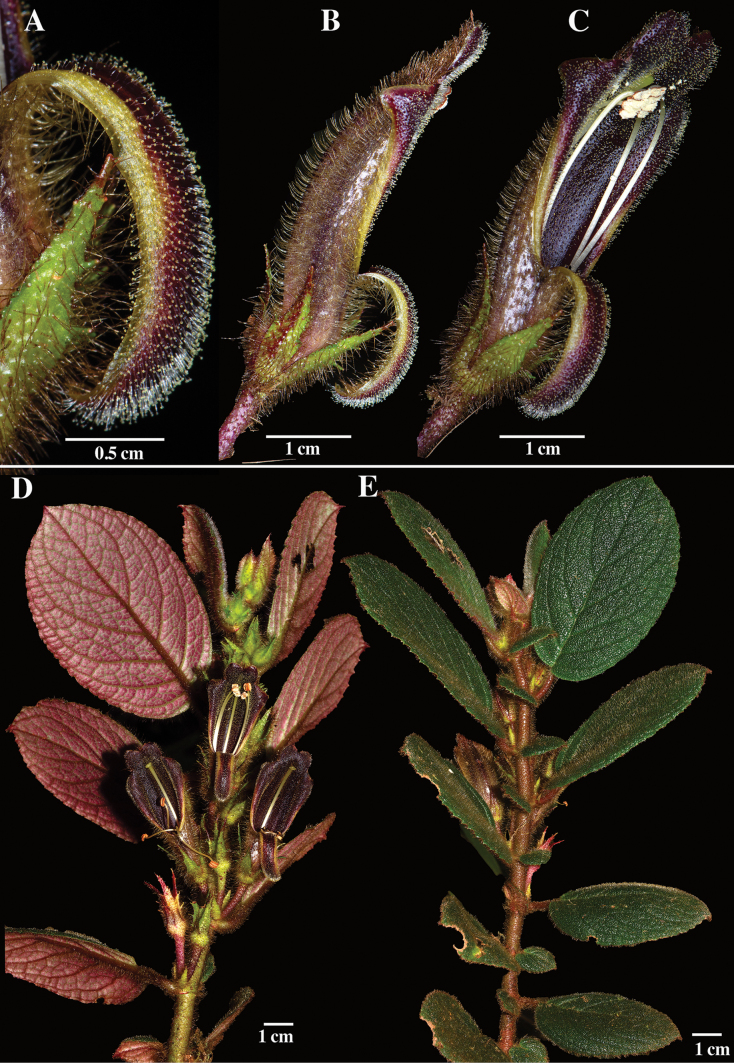
*Columneagolondrinensis* J.L.Clark **A** recurved lower lip of bilabiate corolla **B, C** lateral views of flower **D** abaxial surface of flowering shoot **E** adaxial surface of flowering shoot (**A–E** from *J.L. Clark et al. 18185*). Photos by J.L. Clark.

**Figure 2.**
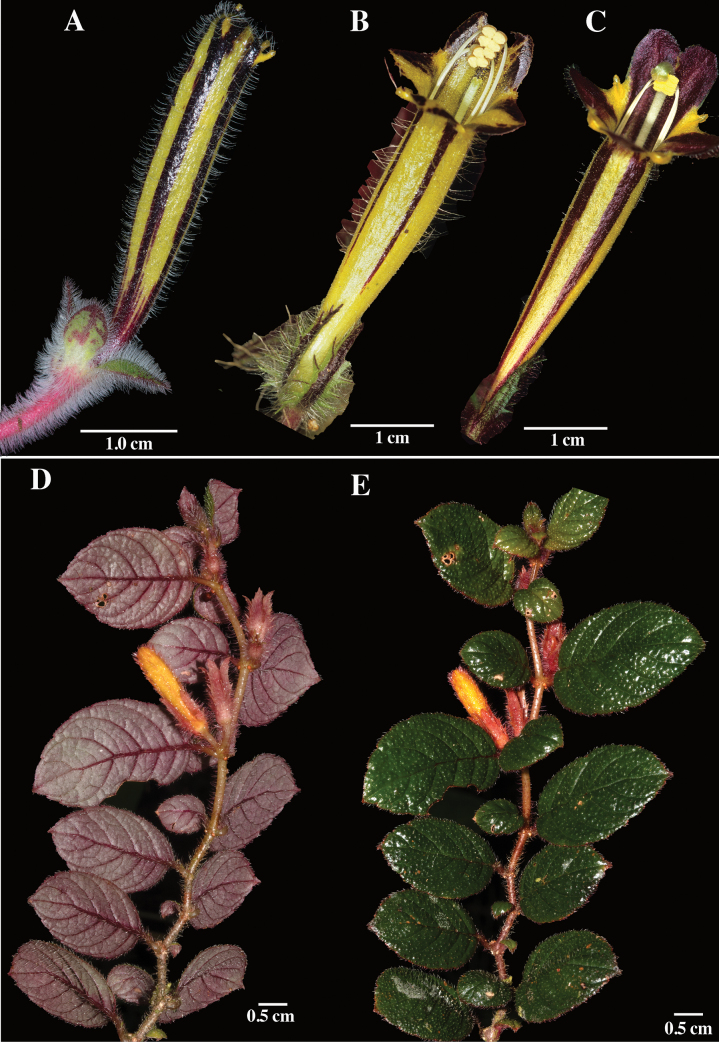
**A***Columneaformosa* (C.V.Morton) C.V.Morton **B***Columneaillepida* H.E.Moore **C***Columneapurpureovittata* (Wiehler) B.D.Morley **D, E***Columneasuffruticosa* J.F.Sm. & L.E.Skog (**A** from *J.L. Clark et al. 19154***B** from *J.L. Clark et al. 9500***C** from *L. Jost 3224***D, E** from *J.L. Clark et al. 19448*). Photos **A, B, D** and **E** by J.L. Clark. Photo **C** by Lou Jost.

**Figure 3.**
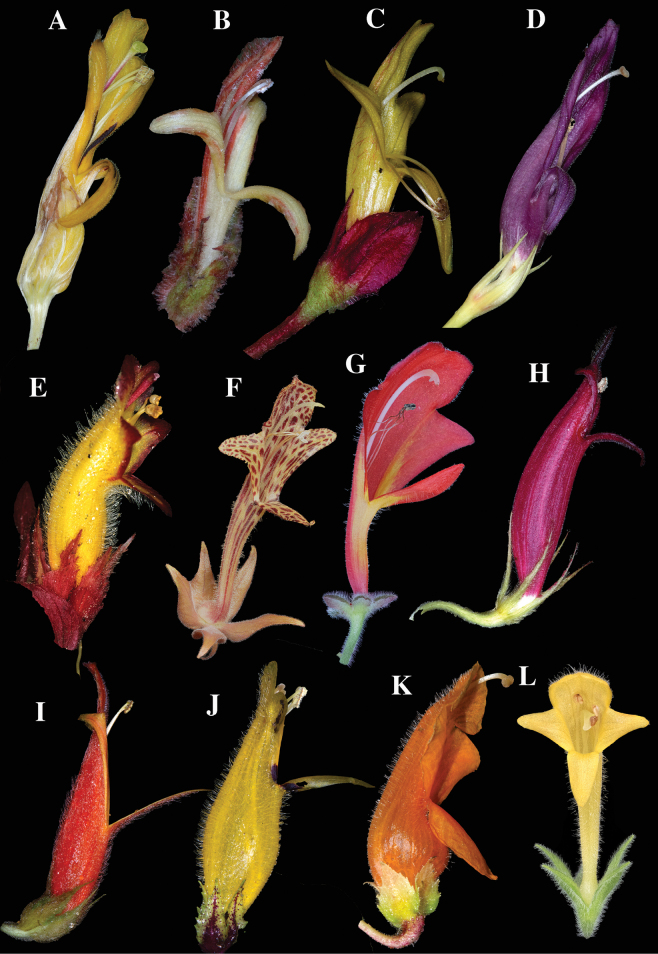
Examples of bilabiate corollas in *Columnea***A***Columneakarsteniana* R.Kr.Singh **B***C.fawcettii* (Urb.) C.V.Morton **C***C.tincta* Griseb. **D***C.stilesiana* M.Amaya & L.P.Kvist **E***C.eubracteata* Mansf. **F***C.schiedeana* Schltdl. **G***C.florida* C.V.Morton **H***C.ferruginea* J.F.Sm. & J.L.Clark **I***C.ceticeps* J.L.Clark & J.F.Sm. **J***C.kucyniakii* Raymond **K***C.strigosa* Benth. **L***C.hirsuta* Sw. (**A** from *J.L. Clark 13159*, **B** from *J.L. Clark 11321*, **C** from *J.L. Clark 12775*, **D** from *J.L. Clark 19470*, **E** from *J.L. Clark 7686*, **F** from *J.L. Clark 18639*, **G** from *J.L. Clark 17645*, **H** from *J.L. Clark 19439*, **I** from *J.L. Clark 17737*, **J** from *J.L. Clark 16303*, **K** from *J.L. Clark 17611*, **L** from *J.L. Clark 17737*). Photos **A–E, H–K** by J.L. Clark. Photos **F, G** and **L** by Wade Collier.

Corolla lobes in *Columnea* are always fused and either shallowly bilabiate or nearly radial (Fig. 2C) to bilabiate (Figs 1, 3). The more common form of bilabiate corolla tubes in *Columnea* is shallowly bilabiate (Fig. 3E, F, H, L) where the lower lip is about the same size as the lateral lobes. The least common form of bilabiate corolla tubes in *Columnea* is deeply bilabiate (Fig. 3A–D) where the lower lip is half the length of the lateral lobes. The corolla tube in *C.golondrinensis* corresponds to deeply bilabiate (Fig. 1), the least common corolla form in *Columnea*. Some examples of *Columnea* with deeply bilabiate corollas include *Columneakarsteniana* R.Kr.Singh (Fig. 3A), *C.fawcettii* (Urb.) C.V.Morton (Fig. 3B), *C.tincta* Griseb. (Fig. 3C), *C.stilesiana* M.Amaya & L.P.Kvist (Fig. 3D), and *C.strigosa* Benth. (Fig. 3K). The presence of dense clusters of glandular trichomes covering the inner surface of the corolla (especially the lower lip) in *C.golondrinensis* (Fig. 1A) is also relatively uncommon in *Columnea*. Two species with glandular trichomes on the lower corolla lip include *C.karsteniana* (Fig. 3A) and *C.stilesiana* (Fig. 3D), but the glandular trichomes are less apparent and more sparsely distributed. The combination of glandular trichomes on the inner corolla surface (Fig. 1A), deeply bilabiate corollas (Fig. 1B, C), dark purple corollas (Fig. 1), and ovate leaves with rust-colored multicellular hispid indumentum (Fig. 1D, E) are unknown in any other species of *Columnea*. The combination of these characters are remarkable and support *C.golondrinensis* as unique and distinct from all other congeners.

## Supplementary Material

XML Treatment for Columnea golondrinensis

## ﻿Additional information

### Author ORCIDs

John L. Clark https://orcid.org/0000-0002-1414-6380

### Data availability

All of the data that support the findings of this study are available in the main text.
